# Immune-checkpoint inhibitor-induced bullous pemphigoid in patients with metastatic renal cell carcinoma: two case reports and systematic review of the literature

**DOI:** 10.2340/1651-226X.2026.46088

**Published:** 2026-09-09

**Authors:** Panagiotis Filis, Magnus Lindskog, Philip Curman, Natalia Kuzmina, Åsa Krantz, Katerina Chatzidionysiou, Fernanda Costa Svedman, Ioannis Zerdes

**Affiliations:** a Department of Oncology-Pathology, Karolinska Institutet, Stockholm, Sweden; b Department of Pelvic Cancer, Genitourinary Oncology, Karolinska University Hospital, Stockholm, Sweden; c Department of Immunology, Genetics and Pathology, Uppsala University, Uppsala, Sweden; d Dermato-Venereology Clinic, Karolinska University Hospital, Stockholm, Sweden; e Dermatology and Venereology Section, Department of Medicine, Karolinska Institutet, Stockholm, Sweden; f Department of Medical Epidemiology and Biostatistics, Karolinska Institutet, Stockholm, Sweden; g Department of Rheumatology, Theme Inflammation and Geriatrics, Karolinska University Hospital, Stockholm, Sweden; h Rheumatology Division, Department of Medicine Solna, Karolinska Institutet, Stockholm, Sweden; i Theme Cancer, Karolinska University Hospital, Stockholm, Sweden

**Keywords:** Immune-checkpoint inhibitors, immunotherapy, renal cell carcinoma, immune-related adverse events, bullous pemphigoid

## Abstract

**Background and purpose:**

Immune-checkpoint inhibitors (ICIs) improve outcomes in renal cell carcinoma (RCC) but may cause immune-related adverse events (irAEs), including cutaneous toxicity. Bullous pemphigoid (BP) is a rare but clinically significant irAE that can require ICI discontinuation.

**Patient/material and methods:**

We report two cases of BP in patients with metastatic RCC treated with ICIs at Karolinska University Hospital, Stockholm, Sweden. In addition, a systematic review was conducted in Medline, Embase, Cochrane Library, and Web of Science to identify published cases of ICI-associated BP in metastatic RCC. Data on patient characteristics, treatment, clinical presentation, diagnostics, management, and outcomes were extracted and descriptively analysed.

**Results:**

Twenty-eight publications describing 30 cases were identified. Median age at BP onset was 69 years (interquartile range [IQR] 65–73), and 86.7% were male. Nivolumab was the most commonly reported ICI monotherapy. Median time from ICI initiation to BP onset was 35 weeks (IQR 16–76). Typical manifestations included tense bullae and vesiculobullous eruptions on the trunk and extremities. Diagnosis was confirmed by skin biopsy and immunological testing in 80% of cases. Management mainly involved corticosteroids, while ICI interruption or discontinuation was required in over half the cases. Both patients from our center improved after immunosuppressive treatment.

**Interpretation:**

ICI-associated BP is a rare but important irAE in metastatic RCC, often occurring after prolonged ICI exposure and potentially requiring treatment modification. Early recognition may improve multidisciplinary management.

## Introduction

The advent of immune-checkpoint inhibitors (ICIs) has revolutionized oncologic therapeutics by reinvigorating the immune system response to malignant cells [1]. By blocking inhibitory molecules such as programmed cell death-1 (PD-1), programmed death-ligand-1 (PD-L1) and cytotoxic T-lymphocyte antigen-4 (CTLA-4), these therapies release anti‐tumor T-cell activity and generate durable responses across a spectrum of solid tumors [2]. Among them, ICIs have markedly enhanced survival outcomes and now represent a therapeutic cornerstone for advanced metastatic renal cell carcinoma (RCC), especially in combination regimens [3]. With the expanding clinical use of ICIs, physicians are increasingly encountering a distinct spectrum of toxicities collectively referred to as immune-related adverse events (irAEs) [4].

IrAEs arise from the broad immune activation and may necessitate treatment modification, ultimately influencing both the continuity and therapeutic efficacy of ICI therapy [5]. Although irAEs may involve virtually any organ system with heterogeneous timing and presentation, cutaneous immune-related adverse events (cirAEs) are among the most frequently observed and are often the earliest to appear [6, 7]. CirAEs comprise a broad range of dermatologic manifestations, with the most frequently reported patterns including maculopapular eruptions, pruritus, psoriasiform changes, eczematous dermatitis, and lichenoid reactions [7, 8]. Bullous pemphigoid (BP) represents an uncommon but clinically significant cirAE, exhibiting distinctive features compared with its idiopathic counterpart, and necessitating heightened vigilance to enable prompt diagnosis and appropriate management [9, 10].

Here, we present two cases of BP emerging in patients with metastatic RCC treated with ICI and further contextualize these observations through a comprehensive systematic review of the literature on immune-checkpoint inhibitor-associated BP (ICI-BP).

## Patients/material and methods

This study comprises two retrospective case reports and a systematic literature review.

### Case reports

The two patients with metastatic RCC included in this report were managed by the authors during their clinical practice at Karolinska University Hospital, Stockholm, Sweden. Clinical data were obtained through a retrospective review of the electronic medical records. Ethical approval was not required for the case reports, according to regulations of the Swedish Ethical Review Authority. Written informed consent for publication was obtained from the patient who was alive at the time of the last follow-up. For the deceased patient, written informed consent for publication was obtained from the patient’s next of kin.

### Search strategy, study selection criteria and data extraction

A systematic literature search was performed in the following databases: Medline (Ovid), Embase (Embase.com), Cochrane library (Wiley), and Web of Science Core Collection (Clarivate). The last search was conducted on 14 June 2024. The search strategy was developed in Medline (Ovid) in collaboration with librarians at the Karolinska Institutet Library. For each search concept, Medical Subject Headings (MeSH-terms) and free text terms were identified. The search was then translated, in part using Polyglot Search Translator, into the other databases [11]. The strategies were proofread by another librarian prior to execution. De-duplication was performed using the method described by Bramer et al. [12]. One final, additional step was added to compare Digital Object Identifiers (DOIs). Full search strategies for all databases are available in the Supplementary Material.

Studies reporting patients with RCC and ICI-related BP (cirAEs) were included in the systematic review. Randomized or non-randomized clinical trials, studies including in vitro and/or *in vivo* experiments, reviews, or previous meta-analyses or studies written in languages other than English were excluded. Data extraction was performed by two investigators (PF, IZ) utilizing a pre-established extraction form. Discrepancies were resolved by a third investigator. The data extraction categories included: author, year, patient characteristics, previous treatments, ICI treatment, ICI regimen (monotherapy or combination therapy), treatment setting, history of autoimmune disease, subsequent oncologic treatment after BP diagnosis, BP presentation characteristics, BP diagnosis, BP treatment, and BP outcomes.

## Results

### Case reports

Case 1: A 60-year-old woman with ECOG performance status 0, RF-positive, anti-citrullinated protein antibodies (ACPA)-negative, non-erosive rheumatoid arthritis (RA) under ongoing treatment with methotrexate, type 2 diabetes mellitus, and hypothyroidism.

At initial presentation, she presented with pain in her left shoulder. Imaging demonstrated a right renal tumor with bilateral pulmonary and multiple bone metastases. Cytological examination of the scapular lesion confirmed metastatic RCC. A CT-guided core biopsy of the renal tumor confirmed clear cell renal cell carcinoma (ccRCC) with the following immunohistochemical (IHC) profile: Vimentin+, CD10+, P504S+, PAX8+, PAX2+, and negative staining for CAIX, RCC, CK7, CK20 and GATA3.

Based on the International Metastatic Renal Cell Carcinoma Database Consortium (IMDC) criteria, her disease was classified as intermediate-risk group. Following multidisciplinary tumor discussion, combination therapy with cabozantinib and nivolumab, rather than nivolumab plus ipilimumab, was recommended because of the patient’s RA, high tumor burden, and symptomatic disease. Palliative radiotherapy (5 Gy × 5 fractions) was administered to the left scapular metastasis. Methotrexate was paused before initiation of ICI therapy, and prednisolone 5 mg/day was initiated.

After three cycles of treatment, radiological reassessment demonstrated complete response of the pulmonary metastases and partial response of the skeletal metastases and primary renal tumor. The patient continued treatment with durable disease control and subsequently underwent deferred cytoreductive nephrectomy one year after treatment initiation. Histopathological examination of the surgical specimen demonstrated complete pathological response without viable tumor cells. Cabozantinib was discontinued after surgery because of impaired renal function, whereas nivolumab monotherapy was continued. Radiological evaluation 6 months after surgery demonstrated stable disease.

Six months after surgery, the patient developed a pruritic skin eruption. Skin biopsy demonstrated lichenoid inflammation consistent with a lichenoid drug eruption. Nivolumab was temporarily withheld, and treatment with oral prednisolone followed by topical clobetasol resulted in partial improvement. After reintroduction of nivolumab, the skin toxicity progressed with the appearance of tense bullae (Figure 1), and nivolumab was permanently discontinued after seven postoperative cycles.

**Figure 1:**
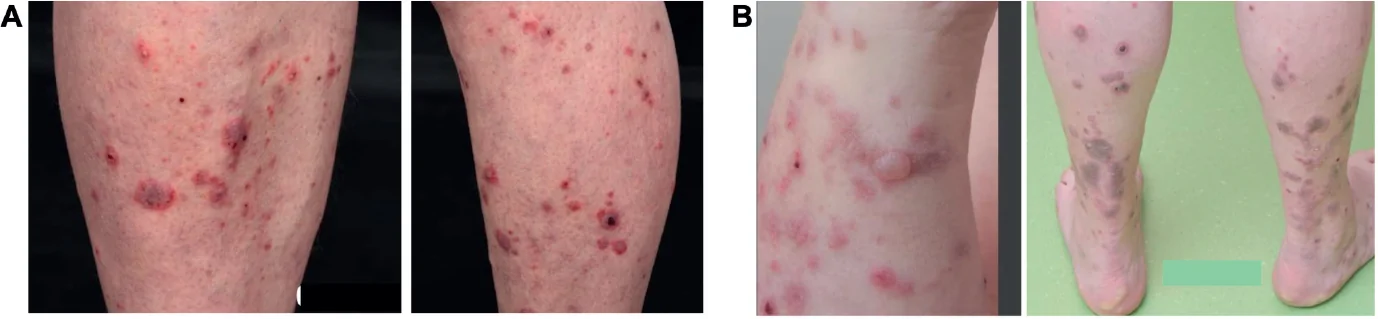
Immune checkpoint inhibitor-associated bullous pemphigoid in Case 1. (A) Pruritic erythematous maculopapular and plaque-like eruption involving the upper extremities and trunk during the initial cutaneous toxicity. (B) Progression to tense fluid-filled bullae on the trunk and extremities after nivolumab rechallenge, leading to permanent discontinuation of immunotherapy.

Further diagnostic work-up demonstrated serum antibodies against BP180, and direct immunofluorescence showed linear deposition of C3 and IgG along the basement membrane, confirming the diagnosis of BP. The patient was treated with low-dose prednisolone, topical corticosteroids, phototherapy, and subsequently mycophenolate mofetil 500 mg twice daily, resulting in complete remission of the skin lesions. At the latest follow-up, there was no evidence of active metastatic disease.

Case 2: A 73-year-old woman with ECOG performance status 0 had a medical history of hypertension, chronic obstructive pulmonary disease, carotid artery stenosis, and previous gallstone disease. She underwent right-sided nephrectomy for ccRCC, Fuhrman grade 3–4, stage pT3 with intravascular tumor thrombus in the renal vein. During follow-up imaging, a progressively enlarging mediastinal lymph node was detected. Endobronchial ultrasound-guided biopsy confirmed metastatic ccRCC with the following IHC profile: CD10+, PAX8+, and negative staining for CK7, TTF1 and P40. Several years after nephrectomy, the patient underwent thoracic surgery; however, the procedure was non-radical due to tumor proximity to major vessels. The patient subsequently received radiotherapy to the mediastinal lesion (5 Gy × 5 fractions). During follow-up, recurrent metastatic disease around the right pulmonary hilum was confirmed bronchoscopically. First-line systemic treatment with axitinib and pembrolizumab was initiated. Shortly after treatment initiation, the patient developed tremors and cognitive symptoms suspected to be treatment-related. Axitinib was temporarily discontinued, leading to resolution of symptoms. Due to recurrent toxicity upon reintroduction of axitinib, the drug was permanently discontinued, and pembrolizumab monotherapy was continued.

The patient initially tolerated pembrolizumab well and maintained good disease control. Thereafter, she developed pruritus and erythematous skin eruptions despite treatment with topical corticosteroids and antihistamines. Intermittent oral ulcerations were also observed. Dermatologic evaluation soon after demonstrated findings suspicious of a pre-bullous blistering disorder. Circulating antibodies against BP180 were detected slightly above the reference range. During the following months, the patient developed tense blisters. Direct immunofluorescence of perilesional skin demonstrated findings consistent with BP. Given the temporal association with ICI therapy, the condition was considered ICI-associated BP, which led to pembrolizumab discontinuation. Following pembrolizumab discontinuation, the patient required hospitalization due to worsening of cutaneous symptoms. She was treated with high-potency topical corticosteroids and systemic antibiotics for suspected secondary infection. Serologic testing confirmed BP180 antibodies. Systemic immunosuppressive therapy with low-dose methotrexate (5 mg weekly) was initiated together with topical corticosteroids and emollients, resulting in gradual clinical improvement.

During the same period, imaging revealed a new metastatic lesion in the right femur with risk of pathological fracture. The patient underwent prophylactic surgery followed by postoperative radiotherapy (8 Gy × 2 fractions).

At dermatologic follow-up the patient showed complete clinical remission of BP. Methotrexate therapy was subsequently discontinued because of mildly elevated liver enzymes, while topical corticosteroids were continued as needed.

Shortly thereafter, the patient was hospitalized due to rapidly declining general condition and severe malignancy-associated hypercalcemia. Despite supportive treatment, her clinical condition continued to deteriorate. Her performance status was considered insufficient for further oncologic treatment, and supportive care was initiated. The patient ultimately died.

### Systematic review

Following deduplication of the records identified from the four databases, 1596 articles were screened by title and abstract, of which 246 underwent full-text review (Supplementary Methods). Overall, 28 articles describing 30 cases of BP associated with ICI in metastatic RCC were included in the systematic review (Supplementary Table 1). The median age at presentation was 69 years (interquartile range [IQR]: 65–73, range 24–89), and most patients were male (26/30, 86.7%) (Table 1). Nivolumab was the most frequently administered ICI regimen (23/30 cases), followed by nivolumab plus ipilimumab (2/30), pembrolizumab (2/30; including one patient treated in the adjuvant setting), atezolizumab (1/30), and axitinib plus sintilimab (1/30). Among the published cases, no patient had a documented pre-existing autoimmune disease before ICI initiation, although three patients developed additional irAEs during treatment (thyroiditis, *n* = 2; diabetes mellitus, *n* = 1). The median time from ICI initiation to BP onset was 35 weeks (IQR: 16–76), although this information was unavailable in four cases (13.3%) (Table 2). Diagnosis was most commonly confirmed by skin biopsy (24/30, 80%), supported by direct or indirect immunofluorescence and, when performed, BP180 and/or BP230 serology. Management mainly included systemic and topical corticosteroids, often combined with immunosuppressive agents or other therapies (Table 2). Most patients improved clinically, although discontinuation of ICI was required in more than half of the reported cases. Information on subsequent oncologic treatment after BP diagnosis was available in only two cases; one patient subsequently received cabozantinib, whereas another continued axitinib after discontinuation of immunotherapy.

**Table 1:** Demographic and clinical characteristics from published case reports.

Characteristics	Number of patients (*N* = 30)
**Demographic**	
Age, median (IQR), years	69 (65–73)
Female, No. (%)	3 (10)
Male, No. (%)	26 (86.7)
Gender not reported, No. (%)	1 (3.3)
**Prior autoimmune disease, No. (%)**	
No history of autoimmune disease	15 (50)
Immune-related treatment events	3 (10)
Not reported	12 (40)
**Treatment setting**	
Metastatic	25 (83.3)
Adjuvant	1 (3.3)
Not reported	4 (13.3)
Treatment prior to ICI, No. (%)*	
Sunitinib	5 (16.7)
Everolimus	3 (10)
Pazopanib	2 (6.7)
Chemotherapy	2 (6.7)
Radiotherapy	1 (3.3)
Sorafenib	1 (3.3)
Trebananib	1 (3.3)
Atezolizumab	1 (3.3)
Not Reported	19 (63.3)
**ICI regimen, No. (%)**	
Nivolumab monotherapy	23 (76.6)
Nivolumab and Ipilimumab	2 (6.7)
Nivolumab (unspecified combination)	1 (3.3)
Pembrolizumab monotherapy	1 (3.3)
Pembrolizumab (unspecified combination)	1 (3.3)
Atezolizumab monotherapy	1 (3.3)
Axitinib and Sintilimab	1 (3.3)
**Type of Response, No. (%)**	
Complete response (CR)	3 (10)
Partial response (PR)	4 (13.3)
Stable disease (SD)	4 (13.3)
Progressive disease (PD)	4 (13.3)
Not reported	15 (50)

ICI: Immune checkpoint inhibitors; BP: bullous pemphigoid; IQR: interquartile range.

* Several studies reported combination of agents. Detailed characteristics are presented in Supplementary Table 1.

**Table 2:** Characteristics and management of the ICI-induced BP.

Characteristics	Number of patients (*N* = 30)
**Time interval between ICI initiation and BP onset**	
Weeks, Median (IQR)	35 (16–76)
Not reported, No. (%)	4 (13.3)
**Prodromal dermatological symptoms, No. (%)**	
Blistering/Erosive lesions	7 (23.3)
Rashes/Erythema	4 (13.3)
Other	2 (6.7)
Not reported	17 (56.7)
Clinical BP features, No. (%)*	
Bullae/Blisters/Vesiculobullous lesions	15 (50)
Pruritus/Itchy eruptions	9 (30)
Erosions	5 (16.7)
Not reported	7 (23.3)
BP localization, No. (%)*	
Extremities	15 (50)
Trunk	13 (43.3)
Face/Head/Neck	5 (16.7)
Oral/Mucosa	3 (10)
Palms/Soles	2 (6.7)
Genitals	3 (10)
Not Reported	5 (16.7)
**BP Grade, No. (%)**	
4	4 (13.3)
3	1 (3.3)
2	4 (20)
1	0 (0)
Not reported	21 (70)
**Skin biopsy, No. (%)**	
Yes	24 (80)
No	0 (0)
Not reported	6 (20)
Histopathological findings, No. (%)*	
Subepidermal blister/separation	17 (56.7)
Eosinophilic infiltrate	15 (50)
Mixed inflammatory infiltrate	9 (30)
Interface/Lichenoid/Spongiotic dermatitis	3 (10)
Immunoglobin/C3 deposition	1 (3.3)
Not reported	10 (33.3)
**Immunofluorescence, No. (%)**	
Direct (DIF) only	19 (63.3)
Indirect (IIF) only	0 (0)
DIF and IFF	4 (13.3)
Not reported	7 (23.3)
**Autoantibodies, No. (%)**	
BP180 positive, BP230 positive	6 (20)
BP180 positive, BP230 negative	4 (13.3)
BP180 positive (without BP230 measurement)	4 (13.3)
BP180 negative (without BP230 measurement)	2 (6.7)
Not reported	14 (46.7)
BP treatment, No. (%)*	
Systemic corticosteroids	18 (60)
Topical corticosteroids	17 (56.7)
Antibiotics	10 (33.3)
Immunosuppressants^1^	6 (20)
Targeted therapy^2^	5 (16.7)
IVIG/Pulse therapy	1 (3.3)
Anti-inflammatory / Non-steroidals	5 (16.7)
Antihistamines / Anti-pruritic	2 (6.7)
Not reported	1 (3.3)
**Adverse events due to BP treatment (%)**	
No adverse events	6 (20)
Hemoglobin decrease due to dapsone	1 (3.3)
Not reported	22 (73.3)
**BP response to treatment, No. (%)**	
Complete resolution	6 (20)
Improvement/Partial resolution	12 (40)
Recurrence after corticosteroid tapering	2 (6.7)
No response	1 (3.3)
Not reported	9 (30)
**Adjustment to ICI therapy, No. (%)**	
Discontinued after BP onset	16 (53.3)
Discontinued due to other adverse event	3 (10)
Continued without interruption	6 (20)
Interrupted after BP onset and re-introduced	2 (6.7)
Treatment already completed prior to BP onset	2 (6.7)
Not reported	1 (3.3)

ICI: Immune checkpoint inhibitors; BP: bullous pemphigoid.

* The clinical presentations of some cases included more than one category.

1 Includes mycophenolate mofetil, methotrexate, cyclosporine, acitretin.

2 Includes rituximab, dupilumab, omalizumab.

## Discussion and conclusion

Reports of BP occurring in the setting of metastatic RCC represent only a small fraction of the already limited literature on this irAE [13]. The two cases presented here, together with the results of our systematic review, draw attention to this uncommon clinical entity, which despite its rarity, carries meaningful implications for ICI treatment continuity and overall patient outcomes. Based on the systematic synthesis of the published cases, the median age of onset of ICI-BP in metastatic RCC was 69 years (IQR 65–73), like that reported for idiopathic BP [14, 15]. In contrast to idiopathic BP, which predominantly affects women, ICI-BP in metastatic RCC demonstrated a marked male predominance (86.7%), likely reflecting the epidemiology of metastatic RCC itself [14, 16]. Although nivolumab was the most frequently reported ICI, BP was also observed during combination immunotherapy and in one patient treated with adjuvant pembrolizumab. Whether BP occurs more frequently during combination therapy than monotherapy cannot be determined, due to the paucity of supporting data and rarity of the condition. Furthermore, among the published cases, no patient had a documented pre-existing autoimmune disease before ICI initiation. Thus, the influence of pre-existing autoimmune disease on the risk of ICI-associated BP remains unknown.

Previous studies have shown a higher frequency of BP in association with PD-1/PD-L1 inhibitors [13]. The mechanism remains incompletely understood but may involve cross-reactivity between tumor and cutaneous basement membrane antigens or loss of immune tolerance following PD-1 blockade [17, 18].

Among the reports describing oncologic outcomes, complete, partial or stable responses were observed more frequently than progressive disease. This is consistent with previous observations that the development of cutaneous irAEs may be associated with improved response to ICI treatment and prolonged survival [19].

The median interval from ICI initiation to BP onset was 35 weeks, somewhat longer than previously reported in mixed cancer cohorts [13]. Interestingly, both of our patients developed BP after prolonged exposure to ICI therapy, illustrating that this toxicity may occur late during treatment.

Bullae and blisters involving the trunk and extremities were the predominant clinical manifestations. Like previous reports, both of our patients experienced a prodromal phase with pruritus and inflammatory skin eruptions before blister formation. In one patient, the initial biopsy demonstrated a lichenoid inflammatory pattern before subsequent histopathology and direct immunofluorescence confirmed BP, highlighting the importance of repeated dermatologic evaluation when clinical suspicion persists.

Skin biopsy together with direct immunofluorescence remained the cornerstone of diagnosis [20, 21]. Systemic and topical corticosteroids were the most frequently used treatments. Current guidelines recommend consideration of steroid-sparing agents such as intravenous immunoglobulin or rituximab in selected patients requiring prolonged immunosuppression [22]. Most patients improved following BP-directed therapy, although discontinuation of ICI was required in more than half of the reported cases. Yet information on post-BP systemic therapy was available for only two patients: one received cabozantinib, while the other continued axitinib after ICI discontinuation. This observation is clinically relevant given the ongoing discussion regarding the optimal duration of ICI therapy in patients with metastatic RCC who respond to treatment, particularly as elective discontinuation after approximately 2 years has recently been suggested not to compromise outcomes in selected patients [23]. The clinical course of our patients further illustrates the challenge of balancing oncologic benefit with management of irAEs, as BP developed after prolonged ICI exposure and required treatment modification or discontinuation.

Certain limitations should be acknowledged. The available evidence is limited to case reports and small case series, precluding firm conclusions regarding incidence, risk factors and treatment outcomes. Moreover, incomplete reporting also resulted in missing clinical data in several published cases.

In summary, BP represents a rare but clinically relevant irAE in patients with metastatic RCC receiving ICI. Our two cases together with the systematic review highlight the heterogeneous presentation of this toxicity, which most commonly occurs after PD-1 inhibition. Although BP frequently requires systemic immunosuppressive treatment and may lead to discontinuation of immunotherapy, most patients respond favorably to dermatologic management. Early recognition of prodromal cutaneous manifestations and close collaboration between oncologists and dermatologists are essential to facilitate timely diagnosis and optimize both dermatologic and oncologic management.

## Supplementary Material



## Data Availability

The data supporting the findings of this study are contained within the article and supplementary materials. Individual patient data from the case reports are not publicly available due to patient confidentiality and privacy considerations.
